# Transformed extracellular vesicles with high angiogenic ability as therapeutics of distal ischemic tissues

**DOI:** 10.3389/fcell.2022.869850

**Published:** 2022-08-31

**Authors:** Nhat-Hoang Ngo, Yun-Hsuan Chang, Cat-Khanh Vuong, Toshiharu Yamashita, Mana Obata-Yasuoka, Hiromi Hamada, Motoo Osaka, Yuji Hiramatsu, Osamu Ohneda

**Affiliations:** ^1^ Laboratory of Regenerative Medicine and Stem Cell Biology, University of Tsukuba, Tsukuba, Japan; ^2^ Department of Obstetrics and Gynecology, University of Tsukuba, Tsukuba, Japan; ^3^ Department of Cardiovascular Surgery, University of Tsukuba, Tsukuba, Japan

**Keywords:** extracellular vesicles, ischemic healing, endothelial progenitor cell, mesenchymal stem cell, hypoxia

## Abstract

**Introduction:** The therapeutic effects of endothelial progenitor cells (EPC) in neovascularization have been suggested; however, to date, few studies have been conducted on the ability of EPC-derived extracellular vesicles (EV) to rescue the ischemic tissues. In order to examine the functional sources of EV for cell-free therapy of ischemic diseases, we compared the functions of EPC-EV and those of Wharton’s Jelly-derived mesenchymal stem cell (WJ-EV) in the flap mouse model.

**Results and conclusion:** Our results demonstrated that in the intravenous injection, EPC-EV, but not WJ-EV, were uptaken by the ischemic tissues. However, EPC-EV showed poor abilities to induce neovascularization and the recovery of ischemic tissues. In addition, compared to EPC-EV, WJ-EV showed a higher ability to rescue the ischemic injury when being locally injected into the mice. In order to induce the secretion of high-functional EPC-EV, EPC were internalized with hypoxic pre-treated WJ-EV, which resulted in a transformed hwEPC. In comparison to EPC, hwEPC showed induced proliferation and upregulation of angiogenic genes and miRNAs and promoted angiogenic ability. Interestingly, hwEPC produced a modified EV (hwEPC-EV) that highly expressed miRNAs related to angiogenesis, such as miR-155, miR-183, and miR-296. Moreover, hwEPC-EV significantly induced the neovascularization of the ischemic tissues which were involved in promoting the proliferation, the expression of VEGF and miR-183, and the angiogenic functions of endothelial cells. Of note, hwEPC-EV were highly uptaken by the ischemic tissues and showed a greater effect with regard to inducing recovery from ischemic injury in the intravenous administration, compared to EPC-EV. Therefore, hwEPC-EV can be considered a functional candidate for cell-free therapy to treat the distal ischemic tissues.

## Introduction

Ischemia is associated with numerous clinical disorders, such as stroke, acute heart failure, acute kidney injury, diabetes, and ischemic wound. Recovery from ischemia requires the initiation of the tissue regenerative process, in which neovascularization is essential (Samura et al., 2017; Smart, 2017).

Numerous reports have shown the potential roles of endothelial progenitor cells (EPC) in the acceleration of neovascularization (Pittenger et al., 2019; Wang et al., 2020). However, the clinical application of EPC as cell therapy has raised several concerns such as maldifferentiation, cell viability, tumorigenicity, or immunorejection (Salybekov et al., 2021; Terriaca et al., 2021). In addition, it has been reported only a limited number of administered tissue stem/progenitor cells located in the damaged tissues, suggesting the functions of these cells were based on paracrine effects (Casado-Diaz et al., 2020; Phinney and Pittenger, 2017). Therefore, to overcome the risks of cell therapy, recent studies have shifted to the application of tissue stem/progenitor cell-derived secretome as cell-free therapy (Salybekov et al., 2021).

In cell–cell communication, extracellular vesicles (EV) have been identified as key mediators in delivering messages between cells (Mathieu et al., 2019; Raposo and Stahl, 2019). EV secreted from stem/progenitor cells can transfer their cargo and modify the phenotypes and behaviors of target cells, which is an ideal source for cell-free therapy (Mathieu et al., 2019; Raposo and Stahl, 2019). Several studies have reported that EPC-derived EV (EPC-EV) promoted angiogenesis in cutaneous wound healing (Li et al., 2016; Zhang et al., 2016; Bai et al., 2020). Of note, EPC-EV shows a high ability to locate in ischemic sites (Salybekov et al., 2021) which might be an ideal source of EV for the treatment of distal damaged organs in patients with conditions such as myocardial infarction, ischemic kidney injury, and stroke. However, whether EPC-EV induces neovascularization and recovery from ischemic injury has not been examined.

In the present study, the ability of EPC-EV to rescue the ischemic tissues was examined using flap mice as a model of ischemic injury (Ichioka et al., 2002; Tepper et al., 2002; Lyu et al., 2022). In addition, EPC were modified to induce the secretion of a transformed EPC-EV (hwEPC-EV) with high abilities in neovascularization for the treatment of the distal ischemic tissues by systematic administration.

## Materials and methods

### Isolation and culture of EPC and Wharton’s Jelly-derived mesenchymal stem cell

Human sample collection and relevant experiments were performed after obtaining informed consent from the donors and were approved by the Ethics Committee of the University of Tsukuba.

EPC were isolated from umbilical cord blood, as previously described (Nagano et al., 2007). Briefly, umbilical cord blood samples were obtained from healthy donors (*n* = 4, female, average age of 32) from the Department of Obstetrics and Gynecology, University of Tsukuba Hospital. The umbilical cord blood was diluted with PBS (1:4) and then mixed with HetaSep solution (StemCell Technologies, Vancouver, BC) at a ratio of 4:1. After that, the mixture was centrifuged at 300 × g for 5 min at 4°C. The upper layer was collected and added RosettedSep solution at a ratio of 20:1 (StemCell Technologies). Then, cells were added to a density gradient buffer Histopaque 1083 g/cm^3^ (Sigma-Aldrich, St Louis, MO) and centrifuged at 1,200 × g for 20 min. The cell layer between the plasma and buffer containing EPC was collected and cultured in an IMDM medium, with 1% Pen-Strep, 10% FBS, 2 mg/ml L-glutamin, and 5 ng/ml bFGF. The isolated EPC was examined the EPC-specific markers, including FITC-anti-human CD31 (303,104, BioLegend, San Diego, CA, United States ), FITC-anti-human CD105 (323,204, BioLegend), APC-anti-human CD133 (397,906, BioLegend), APC-anti-human CD34 (343,608, BioLegend), and APC-anti-human CD45 (982,304, BioLegend) by a flow cytometry (SH800S Cell Sorter, Sony Biotechnology).

To isolate WJ-MSC, human umbilical cord samples were obtained from healthy donors (*n* = 4, female, average age of 32), who were undergoing cesarean section in the Department of Obstetrics and Gynecology, University of Tsukuba Hospital, Tsukuba, Japan. Human umbilical cords were rinsed with cold phosphate-buffered saline (PBS), then the blood clots and blood vessels were removed. After that, the Wharton’s Jelly was collected and cut into 1–2 mm pieces followed by the incubation with 0.1% Collagenase solution at 37°C for 30 min. Then, the digested solution was centrifuged at 1,200 × g for 7 min and washed with PBS. The pellet was collected and added to a culture dish containing MSC culture medium (Iscove’s modified Dulbecco’s medium (IMDM) (Thermo Fisher Scientific, Carlsbad, CA, United States) with 10% FBS (Fetal Bovine Serum, Corning, Lot. 01,421,001), 2 mg/ml l-glutamine (Thermo Fisher Scientific), 5 ng/ml human basic-FGF (Peprotech, London, United Kingdom), and 0.1% (v/v) penicillin-streptomycin (100 U/mL penicillin, 0.1 mg/ml streptomycin; Thermo Fisher Scientific) at 37°C in 5% CO_2_ and a humidified atmosphere for 5 days. The isolated WJ-MSC were examined the MSC-specific biomarkers, including FITC-anti-human CD90 (328,108, BioLegend), APC-anti-human CD73 (344,006, BioLegend), PE-anti-human CD166 (343,904, BioLegend), PE-anti-human HLA-DR (307,606), and APC-anti-human CD45 (982,304, BioLegend) by a flow cytometry (Supplementary Figure S1A). Frozen cell stocks were prepared using Cell Banker solution (ZENOAQ, Koriyama, Japan) and stored in liquid nitrogen for further experiments.

For endothelial cell (EC) culture, human Umbilical Vein Endothelial Cell (HUVEC) was purchased from ATCC and cultured in Endothelial Cell Growth Medium (PromoCell, Heidelberg, DE, Germany), with 1% Penicillin-Streptomycin.

### Isolation and identification of extracellular vesicles

All relevant data of the experiments were submitted to the EV-TRACK knowledgebase (EV-TRACK ID: EV220305) (van Deun et al., 2017). WJ-MSC or EPC derived from passages 4–6 were seeded at a number of 5 × 10^5^ cells in a 10-cm dish and cultured until reaching 70%–80% confluence which corresponded to an average number of 10^6^ cells. Then, the culture medium was removed. Cells were washed with PBS and incubated with a fresh medium containing 0.25% EV-depleted FBS, which showed no apoptotic induction on WJ-MSC and EPC (Supplementary Figure S1B), for a further 24 h to collect conditioned medium (CM). EV-depleted FBS was prepared by ultracentrifugation using the Optima L-XP ultracentrifuge (Beckman Coulter Inc. Brea, CA, United States ) as the instruction of the manufacturer (Beckman Coulter Inc. Brea, CA, United States) and previous studies (Shelke et al., 2014; Kornilov et al., 2018). Briefly, 40 ml of Iscove’s modified Dulbecco’s medium (IMDM) (Thermo Fisher Scientific, Carlsbad, CA, United States) containing 0.25% FBS (Corning^®^, Ref. 35-079-CV, Lot. 01,421,001) was ultracentrifuged at 140,000 × g, 18 h, 4°C with a Beckman Coulter Type 70 Ti Rotor. Then, 30 ml of the supernatant was recovered and used as the culture medium to collect EV.

The derived CM was collected into 50-ml centrifuge tubes at 300 × g for 5 min to remove the cells. The supernatant then was transferred to the new 50-ml centrifuge tubes and centrifuged at 1,200 × g for 20 min to remove cell debris. The supernatant was collected into Beckman Coulter polypropylene tubes (Beckman Coulter Inc. Lot. Z10111SCA, Ref. 326,823) and ultracentrifuged at 140,000 × g for 70 min at 4°C by Beckman Ti70 rotor (Beckman Coulter Inc. S/N 09E678) using the Optima L-XP ultracentrifuge (Beckman Coulter Inc. Brea, CA, United States). After removing the supernatant, the pellet was washed with PBS and ultracentrifuged under the same conditions. Finally, pellets were collected in 100 μl PBS and considered as the isolated EV. The protein concentration of EV was measured using the Bradford assay (Bio-Rad, Hercules, CA). On average, an amount of 120 µg/10^6^ EPC or 80 µg/10^6^ WJ-MSC was collected. The sizes of EV were measured by dynamic light scattering (Zetasizer Nano ZS, Melvern Instruments, United Kingdom).

For hypoxic induction, after reaching the 70%–80% confluence, MSC was incubated at 37°C under 5% CO_2_, and 1% O_2_ for 24 h before collecting the conditioned medium to isolate EV with the same procedure as described above.

### PKH26 labeling of extracellular vesicles

Firstly, the neutralizing buffer was prepared by ultracentrifugation of a volume of 40 ml PBS containing 10% of FBS at 140,000 x g, 18 h, 4°C by a Beckman Coulter Ti70 rotor using an Optima L-XP ultracentrifuge. Then, 30 ml of the supernatant was recovered and used as a neutralizing buffer. For staining, EV with an amount of 120 µg was resuspended in 250 µl of Diluent C (Sigma-Aldrich) and mixed with 250 µl of Diluent C containing 1 µl of PKH26 (Sigma-Aldrich). The EV-dye mixture was incubated in darkness for 5 min and neutralized by a neutralizing buffer. Then, the solution was ultracentrifuged at 140,000 × g for 70 min at 4°C. The pellet was washed with PBS and isolated by ultracentrifugation under the same condition. PKH26 dilution without EV was used as the negative control (Supplementary Figure S1C). The pellet of PKH26-labeled EV was resuspended in 100 µl PBS, and the protein concentration was confirmed again by Bradford assay (Bio-Rad).

### Transmission electron microscopes

An amount of 120 µg of EV was fixed with 2.5% of glutaraldehyde in 0.1 M PBS at 4°C overnight. After that, EV were washed three times with 0.1 M PBS followed by a further fixation with 1% OsO_4_ for 1 hour at 4°C. After fixing with OsO_4_, EV were continued washing three times with 0.1 M PBS, then sequential dehydrating with ethanol and treated with propylene oxide at room temperature for 20 min. Then, the resin osmosis was conducted by treating EV in a mixture of propylene oxide and epoxy resin, at a ratio of 1:1, followed by embedded in Poly/Bed 812 (Polysciences, Warrington, PA, United States) and examined with a JEM-1400 (JEOL, Tokyo, Japan) at 80 kV.

### Internalization of EV to EPC

EPC were seeded at a number of 3 × 10^5^ cells in a 6 cm culture dish then treated with PKH26-labeled WJ-EV at an amount of 12 µg or 24 µg, and cultured for 24 h, followed by washing thrice with PBS. Then, the internalization of WJ-EV to EPC was observed under a microscope (Keyence, BZ-X710) and analyzed using a flow cytometry (SH800S Cell Sorter, Sony Biotechnology), which indicated that treatment of 3 × 10^5^ EPC with an amount of 24 μg PKH26-labeled EV resulted in the internalization of EV to 100% cells (Supplementary Figure S1D).

### Ischemic flap mouse model

Eight-week-old female C57BL/6 mice (Charles River Japan Inc., Yokohama, Japan) were given *ad libitum* access to food and water and maintained in a 12-h light/dark cycle. All animal experiments were approved by the Animal Care Committee of the University of Tsukuba. In this study, the ischemic flap mouse model, a well-established model to examine the neovascularization and the mechanisms involved in ischemic injury (Harder et al., 2014; Wu et al., 2019), was created to examine the functions of EV. The ischemic flap mouse model was performed as described previously (Tepper et al., 2002; Lyu et al., 2022). Briefly, the mice were anesthetized and an incision (3 × 2 cm) was made with a three-sided full skin thickness peninsular flap on the dorsum of the mouse to create an ischemia gradient.

A total of 99 mice were used and 9 mice were divided into each group. EV was injected intradermally at 4 random sites or intravenously *via* tail vein after the surgery at an amount of 60 μg EV/mouse. The ischemic wound healing was observed every day. After 7 days, the necrotic area was measured and the skin tissues were collected for staining. The necrotic area was measured using the ImageJ software program (Schneider et al., 2012) (NIH, Bethesda, MD) based on the necrotic area margin, which was determined by the identical color of necrosis (dark color) (Masson-Meyers et al., 2020). Denote *x*_*i*_ as necrotic area and *y*_*i*_ as incision area, then the percentage of necrotic area *N* is calculated by the following formula:

$$N\;=\;x_{i}/y_{i}\;\times 100\%.$$

### Histological analysis

The skin tissues were fixed overnight with 4% paraformaldehyde, then washed with PBS and soaked in 20% sucrose for 2 h. The wound tissues were embedded in O.C.T compound (Sakura Finetek, Tokyo, Japan) and frozen in liquid nitrogen. The frozen wound tissue sections on the seventh day post-injection were mounted, stained with hematoxylin and eosin (Wako, Osaka, Japan), and observed under a microscope (Olympus, Kyoto, Japan) to assess the tissue structure. Neovascularization was analyzed by immunohistochemical staining of wound tissues with rat anti-mouse CD31 (Lot. 53198; BD Pharmigen), according to the manufacturer’s instructions. The numbers of positive cells were counted in 10 randomly selected fields, and the average number was determined. For PKH26-labeled EV tracking, the tissue sections on the first day post-injection were mounted, stained with DAPI, and observed under a fluorescence microscope (Keyence, BZ-X710). The number of PKH26-positive signals was counted in 30 randomly selected sections per group, and the average number was determined.

### Scratch assay

EPC were seeded at 3 × 10^5^ cells/well in 12-well plates and cultured for 6 h in culture medium for the cell attachment. Then the cells in each well were treated with 24 µg of EV and cultured for 24 h. After washing thrice with PBS, the cells were incubated in a culture medium containing 0.5 μg/ml mitomycin C (Product number 20898-21, Nacalai Tesque, Kyoto, Japan) for 1 h before being scratched. Cells were washed with PBS and incubated at 37°C under 5% CO_2_ for 24 h. Pictures of the scratch gaps were taken at 0, 6, 12, and 18 h under a microscope (Keyence, BZ-X710). The non-migrated areas were analyzed using the ImageJ software program (Ver. 2.1.0) and the percentage of the migrated area was quantified.

### Tube formation assay

EPC or HUVEC was seeded at a number of 5 × 10^5^ cells in a 10 cm cell culture dish and incubated for 6 h in a culture medium for cell attachment. After that, cells were treated with an amount of 40 µg of EV and incubated for 24 h. Cells were detached by trypsin and collected by centrifugation at 300 × g, followed by washing with PBS. Then, a number of 5 × 10^5^ cells/well was seeded in Matrigel-coated 4-well plates and cultured at 37°C for 6 h. To prepare the 4-well plates coated with Matrigel, a volume of 300 µl of growth factor-reduced Matrigel (Corning) was added to each well and incubated at 37°C for 30 min. After 6 h of incubation, cells in Matrigel-coated 4-well plates were observed under a microscope and the images of tubes that formed in 5 randomly selected fields per well were taken. The average tube length was analyzed using the ImageJ software program. The experiments were performed in triplicate.

### Reverse transcription-polymerase chain reaction and quantitative real-time PCR

EPC or HUVEC was seeded at a number of 3 × 10^5^ cells in a 35 mm cell culture dish and incubated for 6 h in a culture medium for cell attachment. Then, cells were treated with an amount of 24 µg of EV and cultured for 24 h. After that, the culture medium was removed, and cells were washed twice with PBS. Next, a volume of 500 µl of Sepasol-RNA I Super G (Nacalai Tesque, Kyoto, Japan) was added to the dish, and cells were harvested in Sepasol-RNA I Super G by cell scrapers for the isolation of total RNA, as the instruction of the manufacturer. To isolate total RNA from EV, EV were incubated with proteinase K (Thermo Scientific, Waltham, MA, United States ) at a concentration of 500 μg/ml for 30 min at 37°C then treated with RNAase A (Thermo Scientific) at a concentration of 0.01 μg/ml for 15 min at 37°C. After that, RNA was isolated by ISOGEN-LS (NIPPON GENE, Tokyo, Japan).

To examine the gene expression, an amount of 1 µg of total RNA was used to synthesize cDNA by an RT-PCR kit (Toyobo Co., Ltd., Osaka, Japan). The expression of the target genes was examined by quantitative PCR (qPCR) using SYBR Green Real-time PCR mastermix (Toyobo, Japan) and analyzed using a GeneAmp 7,500 Fast Real-time PCR System (Applied Biosystems, Waltham, MA). The primer sequences used for the qPCR reactions are listed in Table 1 β-Actin was used as the internal control. In order to examine the expression of miRNAs, 1 µg of total RNA was used to synthesize cDNA by a TaqMan^®^ MicroRNA Reverse Transcription Kit (Applied Biosystems). Then 500 ng of cDNA was used to examine the expression of miRNAs by a GeneAmp 7500Fast Real-Time PCR System (Applied Biosystems) using TaqMan 2✕Universal PCR Master Mix, with AmpErase UNG (Applied Biosystems). The primer sequences used for the analysis of miRNAs expression are listed in Table 2. RNU48 was used as the internal control.

**TABLE 1:** Primers used for quantitative polymerase chain reaction.


Gene name	Sequence	
β-Actin	F	GTG​CGT​GAC​ATT​AAG​GAG​AAG​CTG​TGC
	R	GTA​CTT​GCG​CTC​AGG​AGG​AGC​AAT​GAT
SDF-1	F	AGA​GCC​AAC​GTC​AAG​CAT​CT
	R	CTT​TAG​CTT​CGG​GTC​AAT​GC
HIF1α	F	TGC​TCA​TCA​GTT​GCC​ACT​TC
	R	AAA​ACA​TTG​CGA​CCA​CCT​TC
Ang1	F	AAT​GAG​TTT​ATT​TTT​GCC​ATT​ACC​A
	R	CCC​AGT​GTG​ACC​TTT​TAA​ATA​CAA​C
bFGF	F	GAT​CGA​GCT​CAC​TGT​GGA​GT
	R	CAG​AGT​GTT​GCT​GTG​ACC​AG
CXCR4	F	GGT​GGT​CTA​TGT​TGG​CGT​CT
	R	TGG​AGT​GTG​ACA​GCT​TGG​AG
VEGF	F	AAG​GAG​GAG​GGC​AGA​ATC​AT
	R	ATC​TGC​ATG​GTG​ATG​TTG​GA

**TABLE 2:** Primers used for quantitative polymerase chain reaction of miRNA.


miRNA	Sequence
RNU48	GAT​GAC​CCC​AGG​TAA​CTC​TGA​GTG​TGT​CGC​TGA​TGC​CAT​CAC​CGC​AGC​GCT​CTG​ACC
miR-155	CUC​CUA​CAU​AUU​AGC​AUU​AAC​A
miR-183	UAU​GGC​ACU​GGU​AGA​AUU​CAC​U
miR-296	AGG​GCC​CCC​CCU​CAA​UCC​UGU
miR-210	CUG​UGC​GUG​UGA​CAG​CGG​CUG​A
miR-126	UCG​UAC​CGU​GAG​UAA​UAA​UGC​G

The experiments were performed in triplicate, and the expression levels of the target genes were calculated by using the comparative C_T_ method (2^−ΔΔ*C*^_T_ method). The difference in gene expression in two different samples was calculated by the fold change described as follows:

$${\text{Fold change }=\;2}^{-\Delta {\mathrm{\Delta C}}_{\text{T}}},$$

where

$$\Delta \Delta {\text{C}}_{\text{T}}=\left[\left({{\text{C}}_{\text{T}}\text{ gene of interest }-\text{ C}}_{\text{T}}\text{ internal control}\right)\text{sample}1-\left({\text{C}}_{\text{T}}{\text{ gene of interest }-\text{ C}}_{\text{T}}\text{ internal control}\right)\;\text{sample}\;2\right].$$

### Western blotting

Total protein was extracted from the pellets of cells as described in a previous study (Nagano et al., 2007). Briefly, 30 µg amounts of protein were used for electrophoresis with SDS-polyacrylamide gels and transferred onto PVDF membranes (Merck Millipore, Burlington, MS, United States). After blocking, the membranes were incubated with rabbit anti-Akt and anti-pAkt antibody (9916S, Cell Signaling Technology, Beverly, MA, United States) or rabbit anti-β-actin antibody (GTX109639, GeneTex, Irvine, CA, United States) at 1:1,000 dilution at 4°C overnight.

For EV’ markers, the total protein of EV was extracted using radioimmunoprecipitation assay (RIPA) buffer (Wako), then 50 µg amounts of protein were used for electrophoresis and Western blotting. The membranes were incubated with primary antibodies, including rabbit anti-CD40 (CSB-PA004936LA01HU, Cusabio Tachnology LLC, Houston, TX, United States), rabbit anti-TSG101 (CSB-PA060017, Cusabio Technology LLC), rabbit apolipoprotein A1 (APOA1, GTX40453, GeneTex), or rabbit anti-integrin β1 (GTX128839, GeneTex) at 1:1,000 dilution at 4°C overnight. After that, the membrane was washed and incubated with secondary antibodies, HRP-conjugated goat anti-rabbit IgG (Invitrogen), at a dilution of 1:10,000 at room temperature for 2 h, followed by incubation with chemiluminescence HRP substrate (EMD Millipore) for 1 min. The protein expression was detected using an Image Quant LAS 4,000 System (GE Healthcare, Chicago, IL, United States).

### Statistical analyses

All experiments were performed at least three times. The data are shown as the mean ± SD. Differences were analyzed using the Mann–Whitney U-test of the GraphPad Prism 5 software program (GraphPad Software Inc. San Diego, CA, United States). *p* values <0.05 were considered statistically significant.

## Results

### EPC-EV were uptaken at the injured sites but showed poor ability to rescue the ischemic tissues in the intravenous injection

**FIGURE 1:**
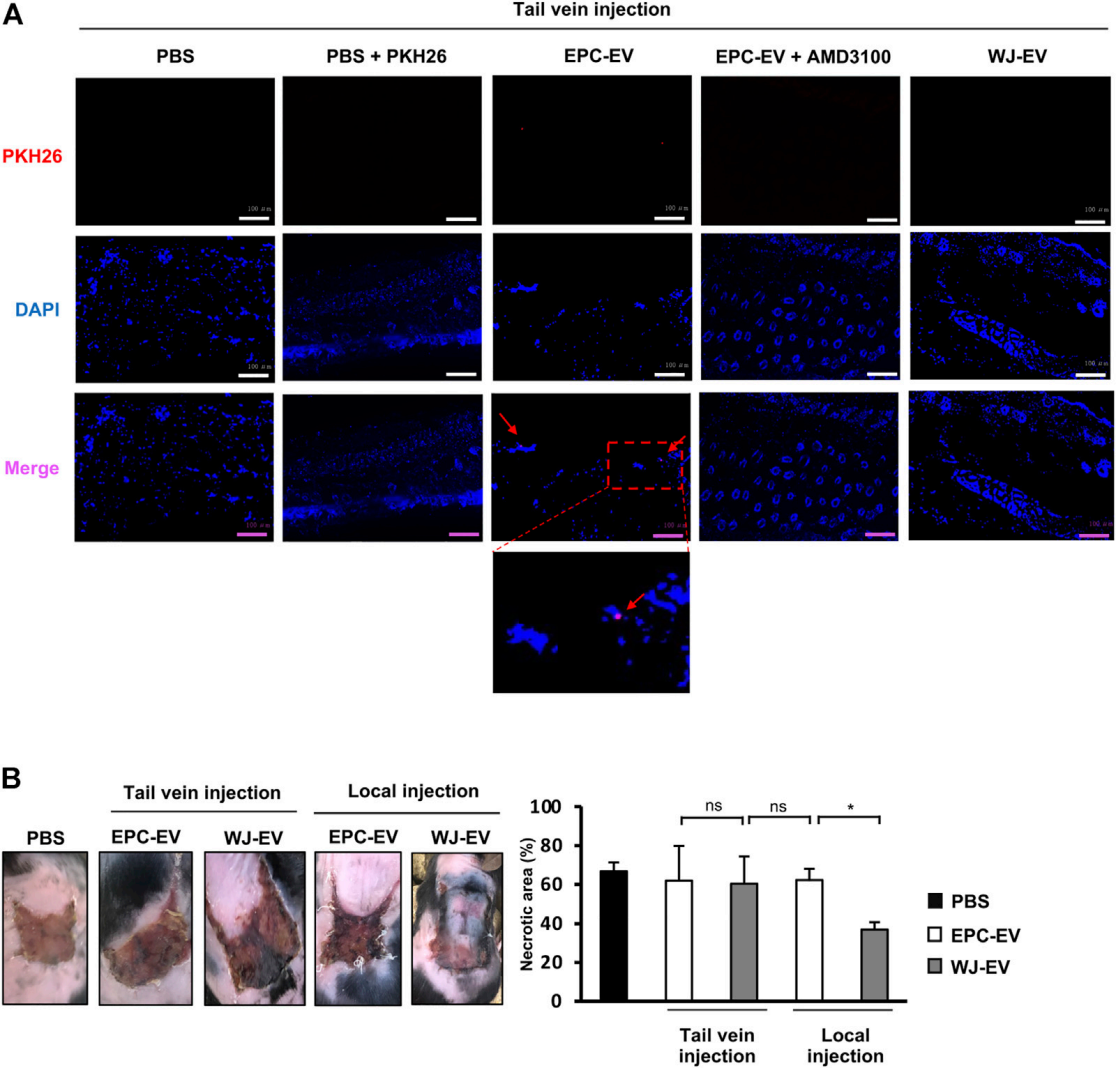
EPC-EV were uptaken at the injured sites but showed poor ability to rescue the ischemic tissues in the intravenous injection **(A)**. The fluorescence imaging analysis of PKH26-labeled EV at 24 h after tail vein injection. EPC-EV + AMD3100: PKH26-labeled EPC-EV treated with CXCR4 antagonist AMD3100. An amount of 60 µg PKH26-labeled EPC-EV in PBS containing AMD3100 (Sigma-Aldrich) at a concentration of 100 nM was injected into the flap mice via tail veins. Photos were captured under a microscope at ×20 magnification. Bars indicate 100 µm (*n* = 3). The experiments were performed triplicated **(B)**. The necrotic area of ischemic flap mice with tail vein and local injection of EPC-EV or WJ-EV (*n* = 9). The data represent the mean ± SD. **p < 0.01, *p < 0.05, ns: no significance.

Our previous studies reported the high migration ability of EPC to the ischemic tissues which is due to the expression of CXCR4 (Tu et al., 2016; Carolina et al., 2018). As EPC-EV contains microvesicles, budding from EPC, we speculated that EPC-EV might possess the expression of CXCR4 which can be recruited by ischemic tissues. Therefore, firstly, we examined the presence of EPC-EV in the ischemic tissues in the intravenous injection. EPC-EV were labeled with PKH26, then injected into the flap mice *via* tail veins. The signals of PKH26 were examined in the ischemic skin tissues after a 24-h injection. As shown in Figure 1A, mice injected with PKH26-labeled EPC-EV showed the signals of PKH26 in the ischemic tissues after 24-h injection, which were not observed in mice injected with PKH26 alone. In addition, mice injected with PKH26-labeled EPC-EV treated with AMD3100, a CXCR4 antagonist, showed no signals of PKH26 (Figure 1A), suggesting the internalization of EPC-EV by the ischemic tissues was involved in CXCR4.

Numerous studies reported the ability of mesenchymal stem cell (MSC)-derived EV to treat ischemia-associated diseases.

Therefore, we further intravenously injected MSC-EV isolated from Wharton’s Jelly tissues (WJ-EV) to the flap mice *via* tail veins and examined their presence in the ischemic tissues. As a result, no signals of PKH26 were observed in the skin tissues of mice injected with PKH26-labeled WJ-EV (Figure 1A), suggesting that WJ-EV were poorly uptaken by the ischemic tissues, in comparison to EPC-EV. Next, we examined the ability of EPC-EV and WJ-EV to treat the ischemic tissues of the flap mice. Surprisingly, although the signals of EPC-EV were observed in the ischemic tissues, the intravenous injection of EPC-EV showed no significant effects to reduce the necrotic area of the ischemic skins (PBS: 68.86%, EPC-EV: 60.71%, Figure 1B), suggesting EPC-EV possess the poor functions to recover the ischemic injury. Meanwhile, in the local injection, WJ-EV showed a higher ability to rescue the flap mice with a smaller necrotic area, in comparison to EPC-EV (WJ-EV: 44.5%, EPC-EV: 64.4%, Figure 1B).

Taken together, these data suggested that EPC-EV were uptaken at the injured sites in the intravenous injection, which is applicable for the treatment of the distal ischemic tissues. However, due to the less able to reduce the necrotic area, it is required to promote the functions of EPC-EV.

### HwEPC, generated by the internalization of EPC with hypoxic WJ-EV, showed the promoted migration and angiogenic functions

Because EV have been identified as a key mediator delivering messages between cells which are applied as a potential tool for cellular modification (Wiklander et al., 2019), we speculated on the ability of WJ-EV to induce the functions of EPC in neovascularization. In addition, hypoxia is a major regulator of neovascularization (Zhang et al., 2018), which has been reported to induce the angiogenic effects of MSC-derived EV on the target cells (Han et al., 2019a; Han et al., 2019b; Almeria et al., 2019). Therefore, we next examined whether the internalization of EPC with WJ-EV or EV isolated from hypoxia pre-treated Wharton’s Jelly-MSC (hWJ-EV) showed the induced effects on the angiogenic ability of these cells.

Firstly, hWJ-EV was isolated and compared to WJ-EV. As a result, hWJ-EV shared similarities with WJ-EV, including the morphology and marker expression, such as positive expression of TSG101, CD40, and integrin β1, and negative expression of apolipoprotein A1 (APOA1) (Supplementary Figures S2A, B). However, in comparison to the nanosize of WJ-EV, hWJ-EV showed a shift change in size distribution (Supplementary Figure S2C), which raised concern about the presence of apoptotic bodies. Therefore, we examined the apoptosis of hypoxia pre-treated Wharton’s Jelly-MSC by staining with Annexin V and 7-AAD. As a result, both Wharton’s Jelly-MSC and hypoxia pre-treated Wharton’s Jelly-MSC showed no apoptotic induction, in comparison to those treated with doxorubicin (Supplementary Figure S2D), suggesting that apoptotic bodies were not contained in hWJ-EV.

**FIGURE 2:**
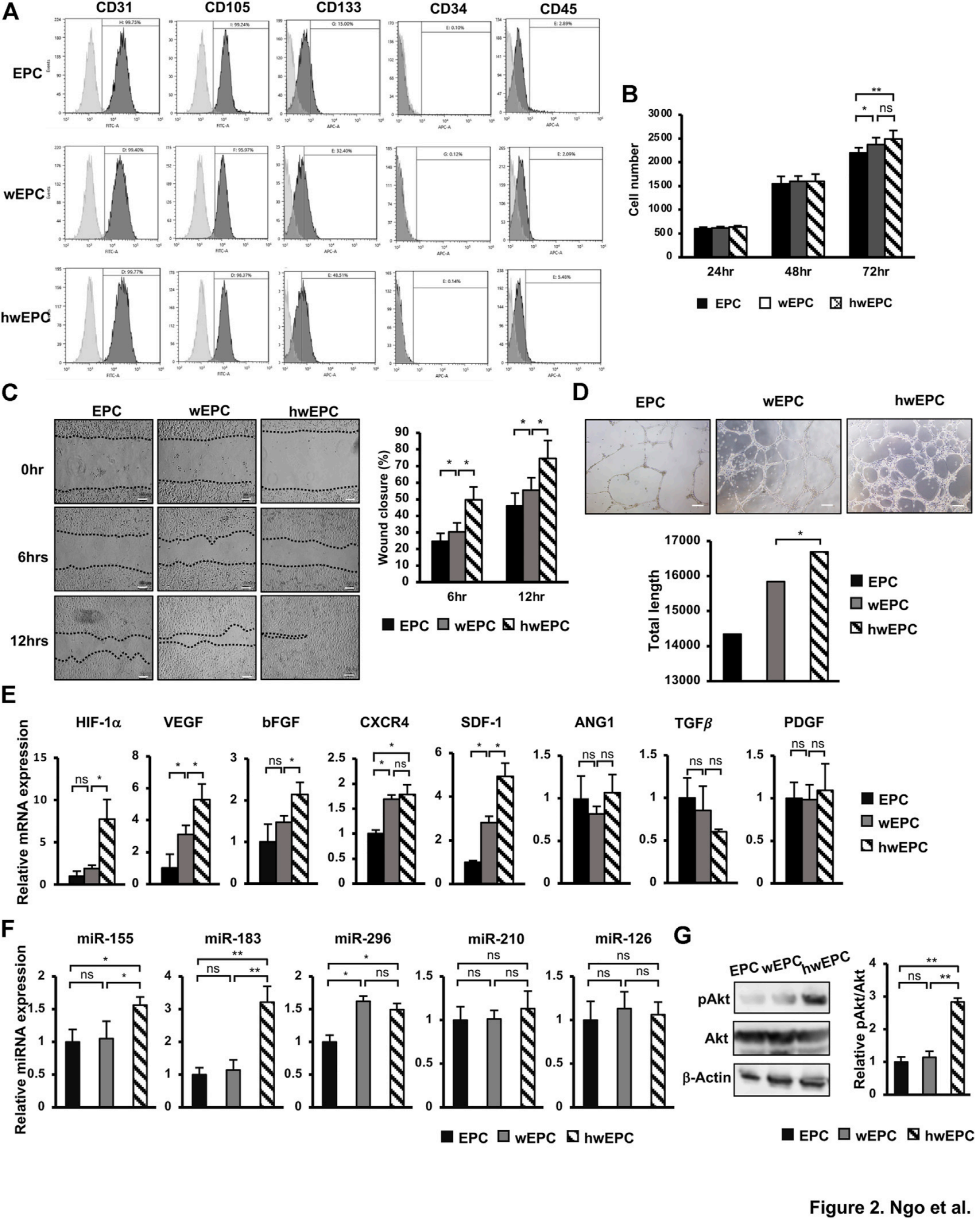
hwEPC showed significantly induced proliferation, migration, and angiogenic abilities, in comparison to EPC and EPC **(A)**. Markers' expression of EPC was examined by flow cytometry **(B)**. The cell proliferation of EPC **(C)**. Migration assay of EPC. Photos were captured under a microscope at 4 × magnification. Bars indicate 100 µm **(D)**. Representative imaging and total length of EPC tube formation. Photos were captured under a microscope at 4 × magnification. Bars indicate 200 µm **(E)**. The gene expression of EPC **(F)**. The miRNAs expression of EPC, normalized with RNU48 **(G)**. The phosphorylation of Akt in EPC was examined by Western Blotting. Full-length Western blots are shown in Supplementary Figure S6. The data represent the mean ± SD. **p < 0.01, *p < 0.05, ns: no significance. N = 3. The experiments were performed in triplicate.

Next, EPC was internalized with PKH26-labeled WJ-EV (wEPC) or PKH26-labeled hWJ-EV (hwEPC), which showed no alteration on the EPC’s specific markers of the recipient cells (Figure 2A). However, hwEPC and wEPC exhibited the promoted proliferation after 72 h of culture, in comparison to the original EPC (Figure 2B). Of note, hEPC showed the significant induction of migration and tube formation abilities in comparison to the original EPC and wEPC (Tube formation: EPC: 14,344.2 ± 1740.1/EPC + WJ-EV: 15,842.4 ± 951.1/EPC + hWJ-EV: 16,691.3 ± 1,104.1) (Figures 2C,D). In addition, hypoxia-inducible factor 1 alpha (HIF-1α), the main factor involved in the promotion of angiogenesis under hypoxic conditions (Zhang et al., 2018), was upregulated in hwEPC (8-fold increase, Figure 2E). Consistently, hwEPC showed the upregulation of target genes of HIF-1α which are multiple critical pro-angiogenic regulators (e.g., VEGF: 5.3-fold increase, bFGF: 2.2-fold increase, and SDF1: 4.9-fold increase, *n* = 3 *p < 0.05, **p < 0.01, Figure 2E).

In addition, we examined the expression of miRNAs that related to the induced angiogenesis in hwEPC, in comparison to wEPC and EPC. As a result, while both wEPC and hwEPC showed the upregulation of miR-296 (Figure 2F), a regulator of VEGF (Feng et al., 2015), in comparison to EPC; only hwEPC exhibited the significantly induced expression of miR-155 and miR-183 (miR-155: 1.5-fold higher, miR-183: 3.2-fold higher, n = 3, *p < 0.05, **p < 0.01, Figure 2F). Previous studies suggested that miR-155 induces the expression of miR-183 (Hu et al., 2014); in addition, miR-183 upregulates the expression of VEGF by the activation of the PI3K/Akt signaling pathway (Zhang et al., 2019; Cao et al., 2020). Therefore, we next examined the activation of Akt in hwEPC. As a result, hwEPC showed the induced phosphorylation of Akt, suggesting the higher activation of the Akt pathway in hwEPC, in comparison to EPC and wEPC (Figure 2G).

Taken together, these data suggested that hwEPC showed promoted proliferation, migration, and tube formation abilities, in comparison to wEPC, which were involved in the upregulation of angiogenic gene expression.

### HwEPC secreted a high functional EV which induced the proliferation and angiogenic functions of endothelial cells (EC) in neovascularization

**FIGURE 3:**
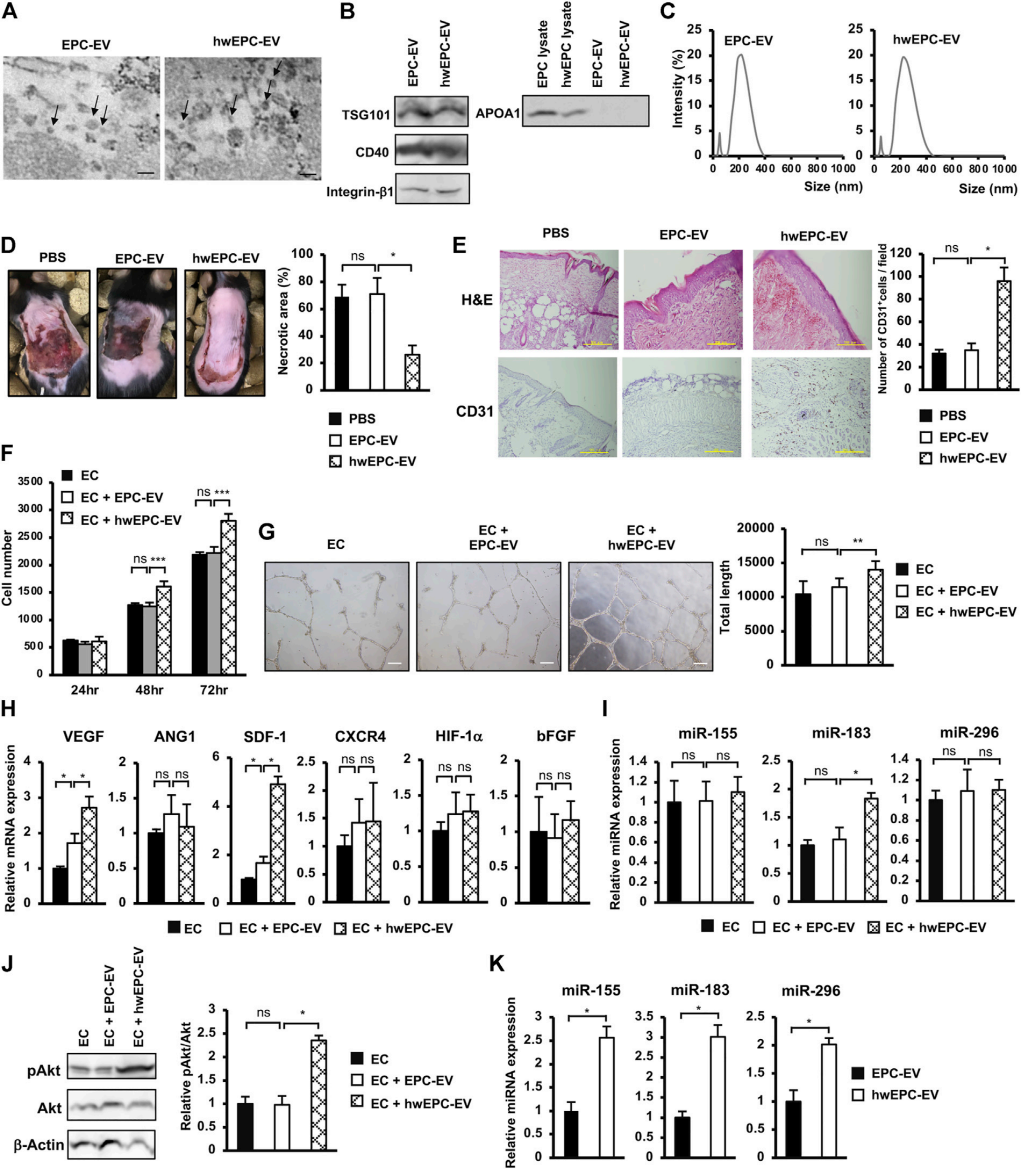
hwEPC secreted a high functional EV which induced the proliferation and angiogenic functions of endothelial cells (EC) in neovascularization **(A)**. Morphology of EPC-EV and hwEPC-EV under a transmission electron microscope. Bars indicate 200 nm **(B)**. Markers expression of EPC-EV and hwEPC-EV. Full-length Western blots are shown in Supplementary Figure S6 **(C)**. Size distribution of EPC-EV and hwEPC-EV. The experiments were performed in triplicate **(D)**. The necrotic area of ischemic flap model mice with local injection (*n* = 9) **(E)**. Immunohistochemical staining of CD31 in skin tissue specimens from locally injected mice. The photos were captured under a microscope at 10 × magnification. Bars indicate 500 µm **(F)**. The cell proliferation of HUVEC (*n* = 3) **(G)**. Representative imaging and total length of HUVEC tube formation (*n* = 3). The photos were captured under a microscope at 4 × magnification. Bars indicate 200 µm **(H)**. The gene expression of HUVEC (*n* = 3) **(I)**. The miRNAs expression of HUVEC, normalized with RNU48 (*n* = 3) **(J)**. The phosphorylation of Akt in HUVEC was examined by Western Blotting. Full-length Western blots are shown in Supplementary Figure S6 (*n* = 3) **(K)**. The miRNAs expression in EPC-EV and hwEPC-EV, normalized with RNU48 (*n* = 3). Data represent the mean ± SD. **p < 0.01, *p < 0.05, ns: no significance. The experiments were performed in triplicate.

Recent studies suggested that EV derived from stem cells is a promising source of cell-free therapy to treat ischemic diseases (Bian et al., 2019). Therefore, we next examined the ability of EV isolated from hwEPC (hwEPC-EV) to rescue the ischemic skin tissues in the flap mouse model, in comparison to the original EPC-EV. Firstly, hwEPC-EV and EPC-EV were characterized which showed morphology of round shape (Figure 3A). In addition, both hwEPC-EV and EPC-EV showed the expression of TSG101, a marker of the exosome, CD40 and integrin β1, the markers of microvesicles, and the negative expression of APOA1 (Figure 3B). These data suggested that hwEPC-EV and EPC-EV consisted of a mixture of exosomes and microvesicles. However, hwEPC-EV showed a shift change in the size distribution, in comparison to EPC-EV (Figure 3C). Therefore, we next examined the apoptosis of hwEPC by staining with Annexin V and 7-AAD. The results showed that no apoptotic induction was observed in hwEPC (Supplementary Figure S3), suggesting that the shift change of the size distribution of hwEPC-EV was not involved in the presence of apoptotic bodies.

Next, hwEPC-EV and EPC-EV were locally injected into the flap mice and examined their ability to induce the recovery of ischemic skin injuries. In comparison to EPC-EV, hwEPC-EV significantly induced the recovery of ischemic skin tissues, in which mice locally injected with hwEPC-EV showed a smaller necrotic area in comparison to those injected with EPC-EV (EPC-EV: 70.97%, hwEPC-EV: 26.33%, Figure 3D). Consistently, the ischemic tissues of mice locally injected with hwEPC-EV showed a higher number of CD31-positive cells, in comparison to those injected with EPC-EV, suggesting that hwEPC-EV significantly induced neovascularization in the ischemic tissues (Figure 3E).

In order to clarify how hwEPC-EV induced recovery at the ischemic sites, we next examined the effects of hwEPC-EV on endothelial cells (EC), a key player in angiogenesis. EC was internalized with the original EPC-EV or hwEPC-EV, then the changes in the phenotypes were examined by analyzing their proliferation and angiogenic abilities. The results showed that in comparison to EPC-EV, hwEPC-EV showed a higher ability to induce the proliferation, tube formation ability, and the expression of angiogenic genes, such as VEGF and SDF-1 in EC (Figures 3F–H).

Next, we examined the expression of miRNAs, which are related to angiogenesis, in EC internalized with hwEPC-EV, to investigate the mechanisms involved in the induced angiogenic abilities of these cells. The results showed that, in comparison to EPC-EV, hwEPC-EV significantly induced the expression of miR-183 in EC (1.8-fold increase, *n* = 3, *p* < 0.05, Figure 3I). Moreover, hwEPC-EV highly activated the Akt signaling pathway in EC, in comparison to EPC-EV (Figure 3J). We next compared the expression of miRNAs in EPC-EV and hwEPC-EV. The results showed that in comparison to EPC-EV, hwEPC-EV showed a higher expression of miR-155, miR-183, and miR-296 (Figure 3K).

Therefore, these data suggested that hwEPC-EV induced the *in vivo* neovascularization and recovery from ischemic injury by promoting the angiogenic functions of EC, which were involved in the induced proliferation and the upregulation of angiogenic genes and miRNA in EC.

### HwEPC-EV were highly uptaken by the ischemic tissues and possess the promoted functions to treat the ischemic tissues

**FIGURE 4:**
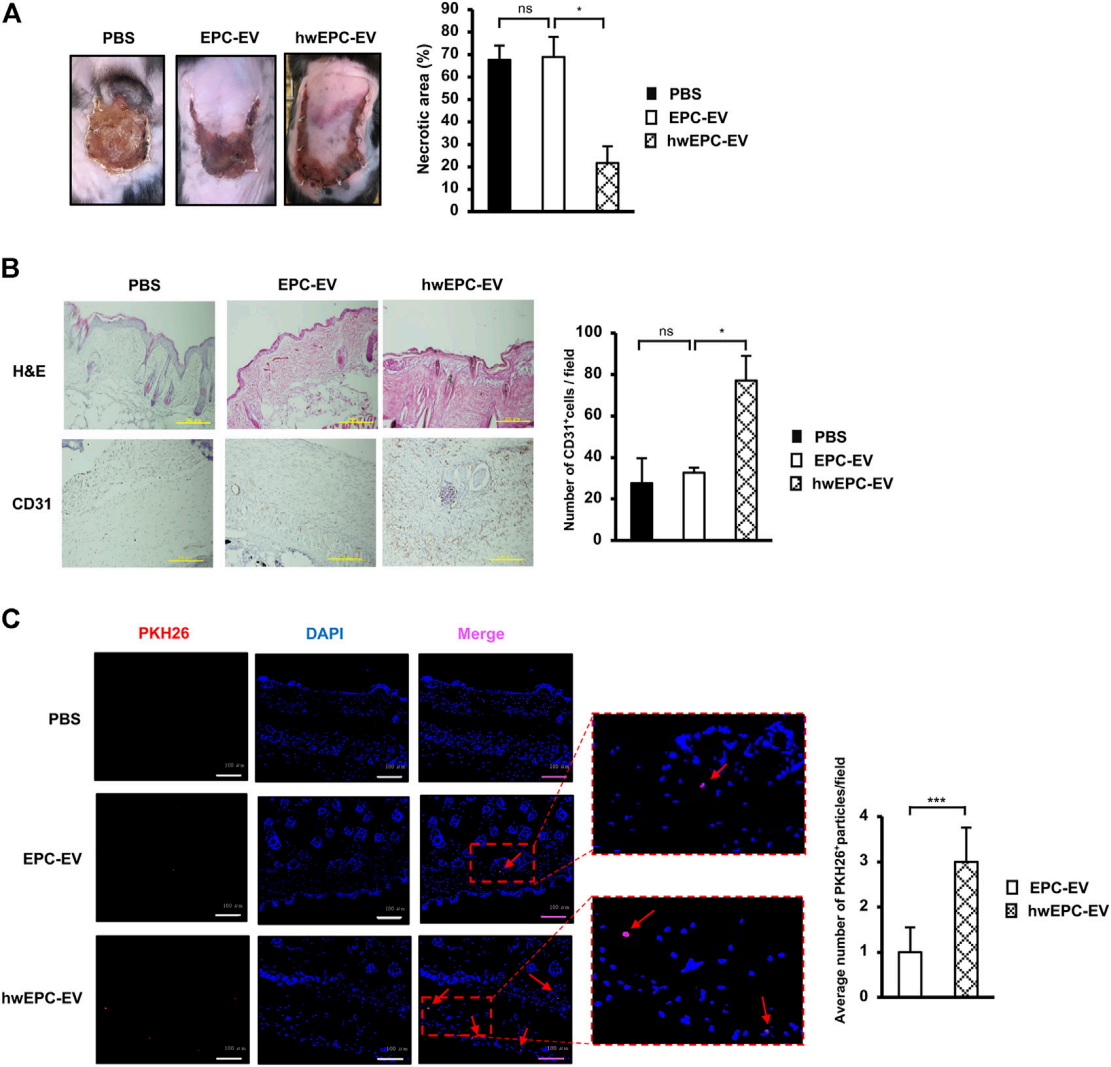
hwEPC-EV were highly uptaken by the ischemic tissues and possess the promoted functions to treat the ischemic tissues **(A)**. The necrotic area of female ischemic flap mice with tail vein injection of EV (*n* = 9) **(B)**. Immunohistochemical staining of CD31 in skin tissue specimens. The photos were captured under a microscope at 10 × magnification. Bars indicate 500 µm **(C)**. The fluorescence imaging analysis of PKH26-labeled EV at 24 h after tail vein injection. Photos were captured under a microscope at 20 × magnification. Bars indicate 100 µm (*n* = 3). The data represent the mean ± SD. **p < 0.01, *p < 0.05, ns: no significance. The experiments were performed in triplicate.

Next, to examine whether the intravenous administration of hwEPC-EV is useful to treat distal ischemic tissues, we injected EV *via* the tail veins of the female flap mice. Surprisingly, similar to the effects of local injection, intravenously injected hwEPC-EV showed a higher ability to reduce the necrotic area of the ischemic skin tissues, in comparison to EPC-EV (PBS: 68%, EPC-EV: 68.9%, hwEPC-EV: 21.6%, Figure 4A). Consistent with the results of the ischemic area, the immunostaining of skin tissues with anti-CD31 antibody suggested that mice intravenously injected with hwEPC-EV showed a higher number of CD31-positive cells in skin tissues compared to those injected with EPC-EV (Figure 4B), which is similar to the effects in the local injection.

In addition, the uptake of EV at the ischemic sites was examined by tracking PKH26-labeled EV under a fluorescence microscope. The results showed that in comparison to the skin tissues of mice injected with EPC-EV, those of mice injected with hwEPC-EV showed a higher number of PKH26 signals (a 3-fold increase of retention PKH26 signals per field, Figure 4C).

In order to examine whether the functions of hwEPC-EV are affected by sex-specific responses, we next conducted the intravenous injection of either EPC-EV or hwEPC-EV to the male flap mice. As a result, in the male flap mice, hwEPC-EV showed a higher ability to induce the healing of the ischemic tissues, in comparison to EPC-EV (PBS: 66.4%, EPC-EV: 66.9%, hwEPC-EV: 17%, Supplementary Figure S4). These results were similar to the effects of hwEPC-EV on the recovery of the ischemic tissues in the female flap mice, suggesting that the functions of hwEPC-EV are independent of the sex-specific responses.

Taken together, these data suggested that hwEPC-EV were highly uptaken at the ischemic sites and showed induced neovascularization, thereby promoting the recovery of the flap mice. Therefore, hwEPC-EV is a promising candidate to develop the treatment of the distal ischemic tissues, associated with numerous diseases, such as myocardial infarction, ischemic kidney injury, and stroke. Further study is still needed to find effective treatments for each tissue.

## Discussion

Tissue stem cells, such as MSC and EPC, are the candidates to treat ischemia-associated diseases. However, several studies suggested the poor homing to the injured sites of MSC due to the low expression of chemotaxis receptors (Ullah et al., 2019). Indeed, we found that in comparison to EPC, WJ-MSC showed a lower expression of CXCR4 (Supplementary Figure S5A), a chemotaxis receptor involved in the migration of cells toward SDF-1 signaling in the ischemic tissues (Wang et al., 2021). In addition, when being intravenously injected *via* the tail veins of the flap mice, WJ-MSC exhibited lower migration (Supplementary Figure S5B) and poor ability to induce the recovery of the ischemic skins (Supplementary Figure S5C), in comparison to EPC.

Interestingly, we found that the ability of EPC to rescue the ischemic tissues is promoted after the intravenous administration, in comparison with the local administration. Mice with intravenous EPC injection showed significant recovery from ischemic injury, in comparison to those with local EPC injection (Supplementary Figure S5C, *p* < 0.01). In consistence with our data, previous studies reported that direct administration of EPC into the ischemic tissues reduces the regenerative capacity and survival of EPC which is involved in the lack of blood flow necessary to deliver oxygen and nutrients to support the long-term survival of engraftment (Goto et al., 2016). Therefore, intravenous injection of EPC to the ischemic tissues through a homing mechanism is an effective approach for the treatment of the distal ischemic tissues, which are located apart from the sites of injection.

However, the application of EPC as cell therapy has been faced with potential risks such as maldifferentiation, cell viability, or tumorigenicity (van Niel et al., 2018). Meanwhile, EV secreted from EPC, which can transfer their cargo and modify the phenotypes and behaviors of target cells, is an ideal source for cell-free therapy to overcome these potential risks (Kong et al., 2021). As EPC-EV are membrane-contained vesicles released by EPC, it is suggested that EPC-EV also express similar receptors to the parental EPC, which are responsible for the ischemic tropism. Indeed, a previous study demonstrated the retention of EPC-EV administered *via* intravenous injection in islet cell transplantation SCID model mice (Bai et al., 2020). In addition, another study of EPC-EV function on the kidney after ischemic injury indicated the uptake of EV in the injury site relates to CXCR4/SDF-1 chemotaxis mechanism (Viñas et al., 2018). Of note, in our study, the tracking analysis of ischemic tissues showed the signals of PKH26-labeled EPC-EV in the intravenous administration (Figure 1A), suggesting the possibility of using EPC-EV for the treatment of distal organs located apart from the injection sites. However, our results showed that the effect of EPC-EV to induce the recovery of the ischemic tissues was not as expected, in which mice injected with EPC-EV remained in the high necrotic area at the ischemic skins (Figure 1B). Thus, the modification of the cargo of EPC-EV is necessary for improving neovascularization and treating distal ischemic sites.

**FIGURE 5:**
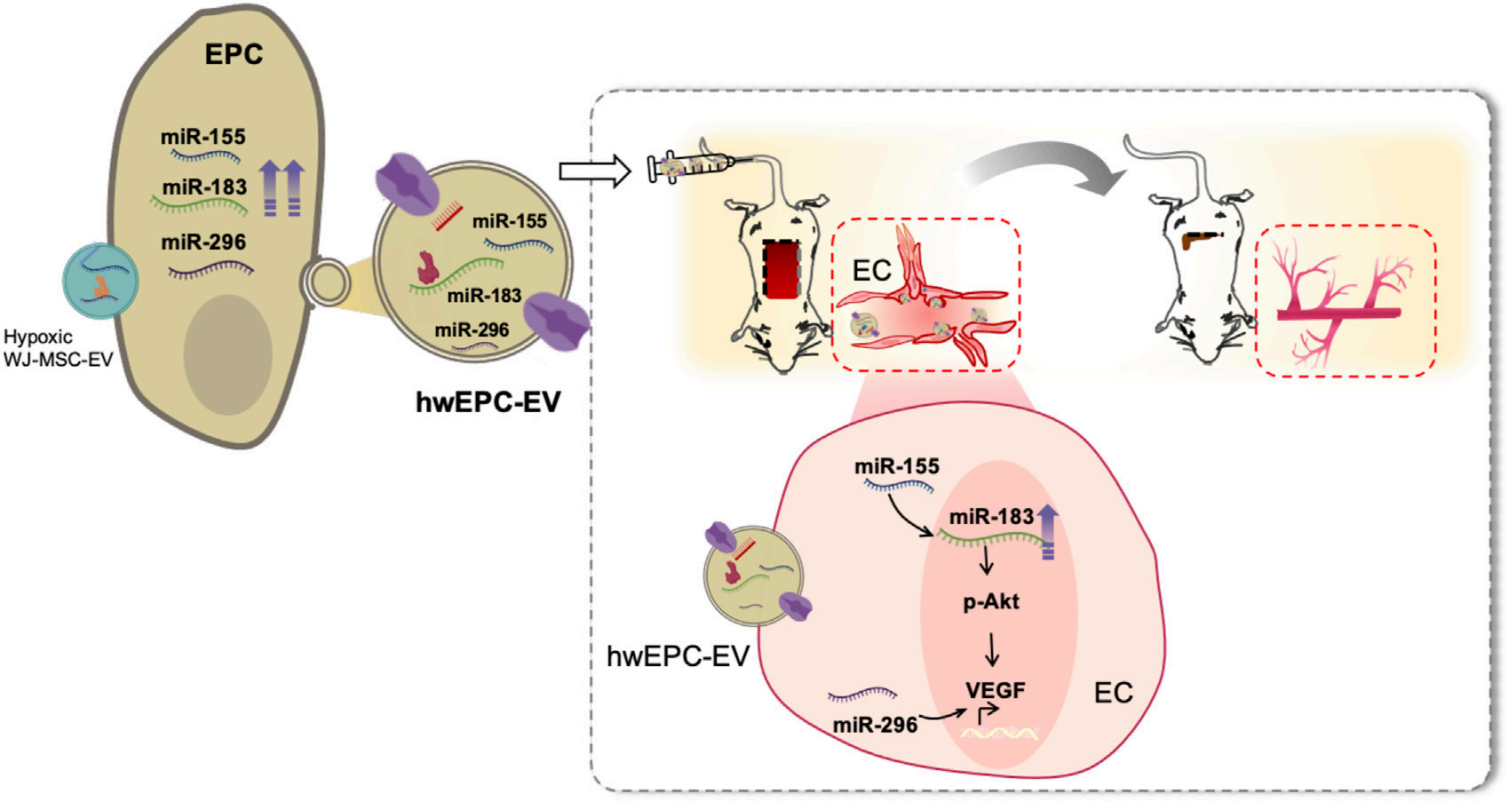
hwEPC-EV is a promising candidate to treat ischemic injuries of the distal organs and tissues. hwEPC-EV is preferentially uptaken at the ischemic tissues of the flap mouse model in the intravenous injection. At the ischemic sites, hwEPC-EV induced the proliferation and angiogenic functions of ECs which might be involved in the transfer of miRNAs related to angiogenesis, including miR-155, miR-183, and miR-296 to EC. Therefore, the intravenous injection of hwEPC-EV resulted in the recovery of the ischemic tissues.

The conventional genetic intervention of parental cells using vectors or viruses has been proven with the successful generation of modified EV (Ma et al., 2018; Wiklander et al., 2019). However, this approach shows limited efficacy, as it is unable to manipulate a combination of genes or pathways. To overcome these limitations, recent advances have used EV (Murphy et al., 2019), which can transfer the biomolecules from the donor cells to the recipient cells, thereby modulating the phenotype and functions of these recipient cells (van Niel et al., 2018; Raposo and Stahl, 2019). Nevertheless, although numerous studies have succeeded in genetically modifying target cells (Trinh et al., 2016; Tu et al., 2016), to date, no report has shown the ability to use EV on parental cells to generate modified EV. In the present study, we found that the internalization of EV derived from hypoxic treated WJ-MSC to EPC resulted in the secretion of a modified hwEPC-EV from isrecipient EPC. In comparison to the original EPC-EV, hwEPC-EV were highly uptaken by the ischemic tissues and showed enhanced neovascularization and ischemic injury repair functions (Figure 5). Our study suggested the novel application of EV in which cargo loading of EV not only modifies the phenotypes of target cells but also promotes the functions of EV derived from these target cells.

In neovascularization, EC is identified as a key player in angiogenesis, which proliferates and forms new vessels from pre-existing vessels (Rodrigues et al., 2019). Our results suggested the ability of hwEPC-EV to promote the proliferation and angiogenic functions of EC, which were involved in the induced neovascularization and recovery of the ischemic injuries *in vivo*. Compared to EPC-EV, hwEPC-EV showed a higher expression of miR-296, miR-155, and miR-183, the regulators of angiogenesis, which might be delivered to EC. miR-296 has been reported to induce the expression of VEGF and angiogenesis in a previous study (Feng et al., 2015). Meanwhile, several studies reported that miR-155 is involved in the upregulation of miR-183 (Hu et al., 2014), which activates the Akt/VEGF signaling pathway (Zhang et al., 2019; Cao et al., 2020). Consistently, EC internalized with hwEPC-EV showed the upregulation of miR-183 and VEGF, and the activation of the Akt pathway. However, no upregulation of miR-296 and miR-155 was observed in EC internalized with hwEPC-EV in qRT-PCR analysis. Therefore, we hypothesized that the number of miRNAs delivered from hwEPC-EV to EC might not be enough to be detected by qRT-PCR, thus it is worth further sequencing analysis of EC internalized with hwEPC-EV. In addition, the effects of knockdown of miR-155 and miR-296 expression on angiogenic functions of EC internalized with hwEPC-EV should be addressed in further studies.

Different uptake mechanisms of EV by the target tissues depending on EV size have been reported in recent studies (Murphy et al., 2019; Xie et al., 2022). In our study, mice intravenously injected with PKH26-labeled hwEPC-EV showed a higher number of PKH26 signals in the ischemic tissues, in comparison to EPC-EV (Figure 4C), which might be involved in the differences in size distribution between hwEPC-EV and EPC-EV (Figure 3C). In addition to the size of EV, another study suggested the important role of surface proteins on the tropism of EV (Kooijmans et al., 2016). Since EV possess the inherent surface proteins from the parental cells, EV with a big size, such as microvesicles budding from the cell membrane, might contain more surface chemotaxis receptors, for instance, CXCR4 (Salybekov et al., 2021). Based on the shift change in size distribution of hwEPC-EV, we hypothesized the possibility that the population of hwEPC-EV might contain more microvesicles than EPC-EV.

Several studies suggested that recycling exists in the endosomal biogenesis of EV, in which the materials of donor EV might be sustained and become a part of EV produced by recipient cells (Grant and Donaldson, 2009; O’Sullivan and Lindsay, 2020; Xie et al., 2022). Our data showed that in comparison to EPC-EV, hwEPC-EV exhibited a higher ability in neovascularization and the shift change in size distribution (Figure 3C). Thus, there is a possibility that hwEPC-EV is a hybrid of hWJ-EV and EPC-EV, which might affect their size distribution. A further study to examine the mechanisms of how hWJ-EV were uptaken and re-generated in hwEPC by specifical characterization each EV *via* membrane receptors analysis, proteomics, mRNA, and miRNA sequencing is necessary to clarify this speculation.

Ischemia is associated with numerous metabolic diseases which require an effective method to treat the ischemic tissues. Although numerous therapies have been developed to reduce inflammation and improve neovascularization of injured tissues to induce recovery from ischemia, effective treatments for distal organs, such as the brain, heart, and kidney, are still limited. Therefore, hwEPC-EV, which was preferentially captured at ischemic sites and highly induced neovascularization, may be a promising candidate for cell-free therapy for the treatment of a wide range of ischemia-associated diseases. However, compared to cell therapy using EPC, our results suggested that although hwEPC-EV promoted the recovery of the ischemic tissues of the flap mice, their ability was lower than EPC administration (Figure 4A and Supplementary Figure S5C). These results might be due to the fact that our present study examined the functions of hwEPC-EV to treat ischemic tissues using the flap mouse model, which is related to the ischemic wound. In the wound healing process, in addition to EV, EPC secretes numerous cytokines, and growth factors, to promote neovascularization. Therefore, the efficacy of hwEPC-EV will need to be fully studied in various animal models of ischemic diseases.

## Conclusion

Our study suggested the novel application of EV in genetic intervention and modification of the target EV. Using hypoxic WJ-MSC-derived EV as a carrier of angiogenic functional cargo loading to EPC, we generated the modified hwEPC-EV that were highly uptaken by the ischemic tissues and showed a high neovascularization function to induce recovery from ischemic injury in the skin flap model. The findings suggest that hwEPC-EV may be a new promising tool for the treatment of distal organs with ischemic injury.

## Data Availability

The original contributions presented in the study are included in the articleSupplementary Material; further inquiries can be directed to the corresponding author.

## Abbreviations

EV, extracellular vesicles; EPC, endothelial progenitor cells; EPC-EV, endothelial progenitor cell-derived EV; WJ-MSC, Wharton’s Jelly-derived mesenchymal stem cells; WJ-EV, EV isolated from WJ-MSC; hWJ-EV, EV isolated from hypoxia-pre-treated WJ-MSC; wEPC, endothelial progenitor cell-internalized with EV from WJ-MSC; hwEPC, endothelial progenitor cell-internalized with EV from hypoxic treated WJ-MSC; hwEPC-EV, extracellular vesicles from hwEPC; EC, endothelial cells.
