# Supplementary material for: Transformed extracellular vesicles with high angiogenic ability as therapeutics of distal ischemic tissues
Source: Front Cell Dev Biol. 2022 Aug 31;10:869850. doi: 10.3389/fcell.2022.869850 (PMC9473158; doi:10.3389/fcell.2022.869850)
Supplement: Supplementary file 1 [file DataSheet1.PDF]

Transformed Extracellular Vesicles with High Angiogenic Ability as Therapeutics of Distal Ischemic Tissues

Ngo Nhat Hoang[1], Yun Hsuan Chang[1], Vuong Cat Khanh[1], Toshiharu Yamashita[1], Mana Obata-Yasuoka[2], Hiromi Hamada[2], Motoo Osaka[3], Yuji Hiramatsu[3], and Osamu Ohneda[1]

[1]Laboratory of Regenerative Medicine and Stem Cell Biology, University of Tsukuba, Tsukuba 305-8575, Japan.

[2]Department of Obstetrics and Gynecology, University of Tsukuba, Tsukuba, Japan.

[3]Department of Cardiovascular Surgery, University of Tsukuba

Address correspondence to: Osamu Ohneda, MD, PhD, Laboratory of Regenerative Medicine and Stem Cell Biology, University of Tsukuba, 1-1-1 Tsukuba 305-8575, Japan.

E-mail: oohneda@md.tsukuba.ac.jp

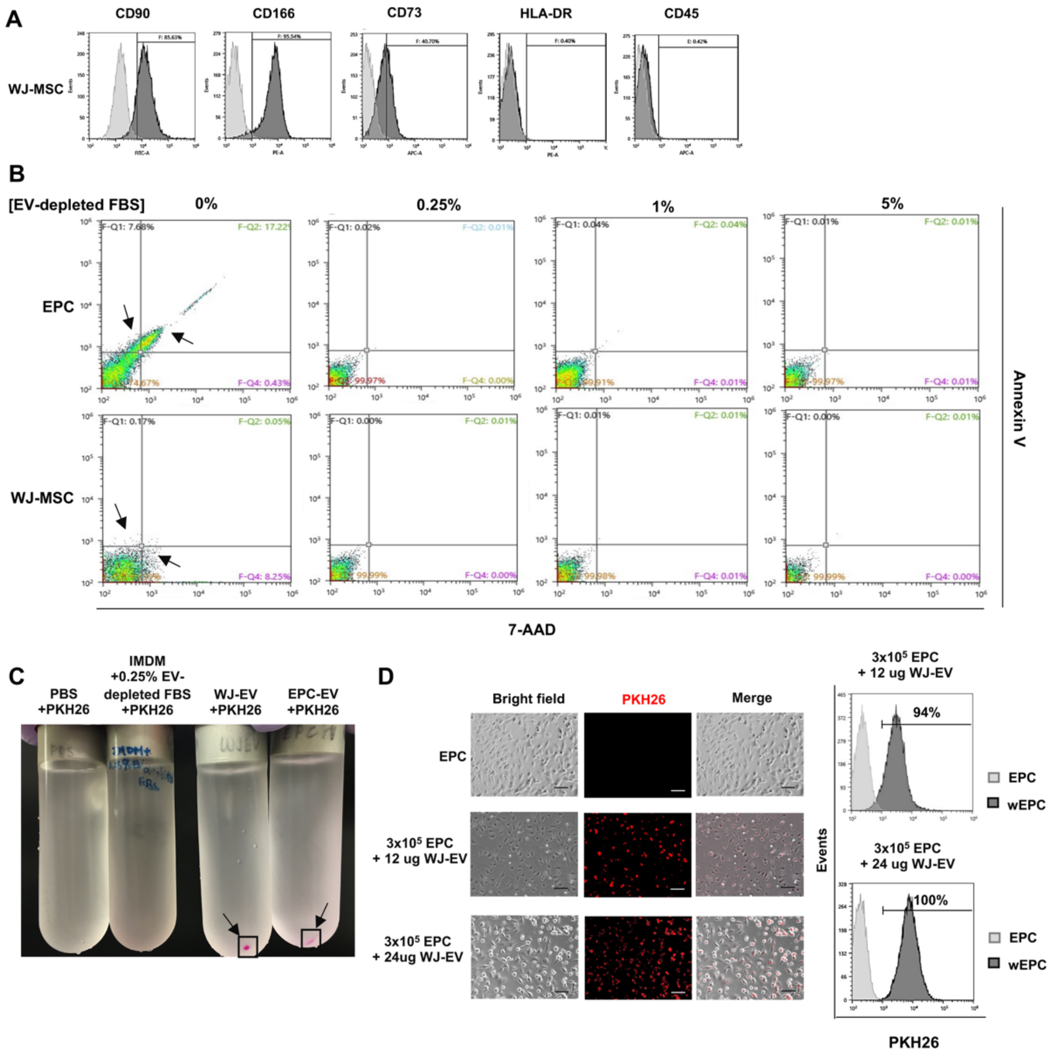

Supplementary Figure 1. A. The MSC-specific markers expression of WJ-MSC. **B.** Apoptosis of EPC and WJ-MSC cultured under IMDM containing EV-depleted FBS at different concentration. The apoptosis of cells was examined by staining a number of 10^6 cells with APC-Annexin V apoptosis detection kit with 7-AAD (640930, BioLegend) as the instruction of the manufacturer. **C.** PKH26-labeled WJ-EV and EPC-EV after ultracentrifugation. Samples of PKH26 in PBS or IMDM containing 0.25% EV-depleted FBS were used as the controls. **D.** The internalization of PKH26-labeled WJ-EV to EPC examined by observation under a fluorescence microscope and analysis by a flow cytometry. Bars indicated 200 μm. All experiments were performed in triplicate.

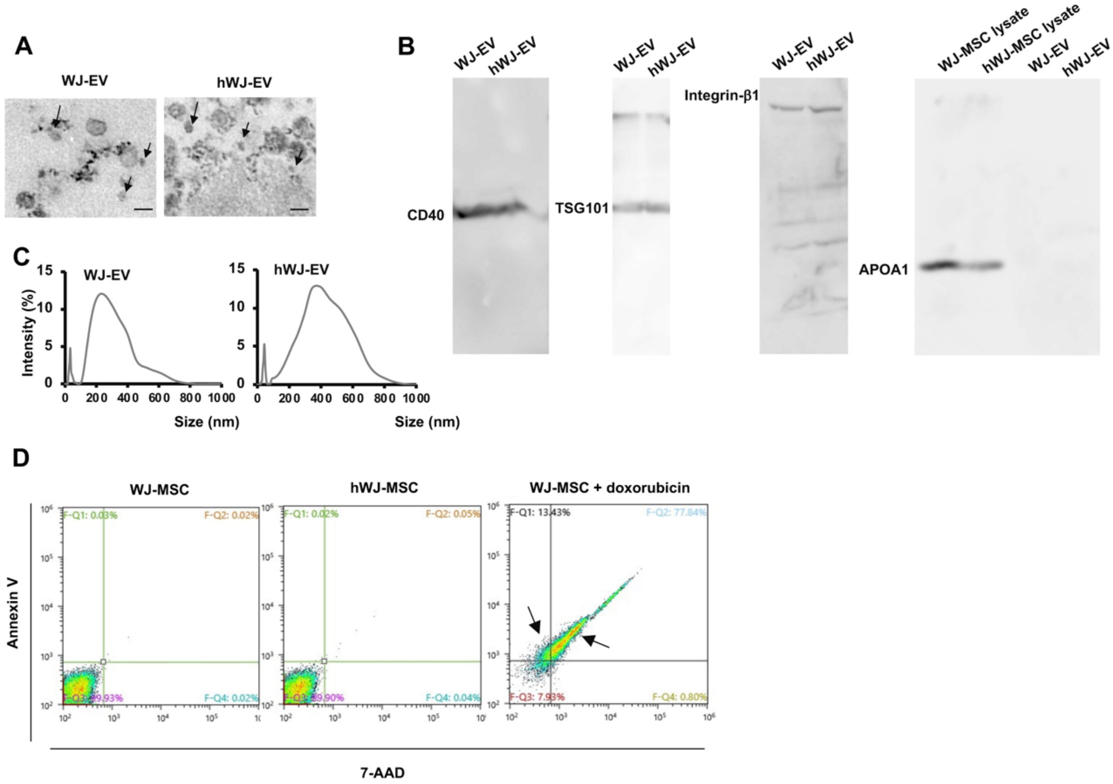

Supplementary Figure 2. A. Morphology of WJ-EV and hWJ-EV examined under a transmission electron microscope. Bars indicated 200nm. **B.** The expression of EV markers in WJ-EV and hWJ-EV examined by Western Blotting. **C.** Size distribution of WJ-EV and hWJ-EV. **D.** The apoptosis of WJ-MSC and hypoxia-pretreated WJ-MSC (hWJ-MSC) was examined by staining a number of 10^6 cells with APC-Annexin V apoptosis detection kit with 7-AAD (640930, BioLegend). WJ-MSC treated with 0.25 μg/mL doxorubicin (Cayman Chemical, Ann Arbor, MI, USA) for 24 hours were used as the control groups with apoptosis. All experiments were performed in triplicate.

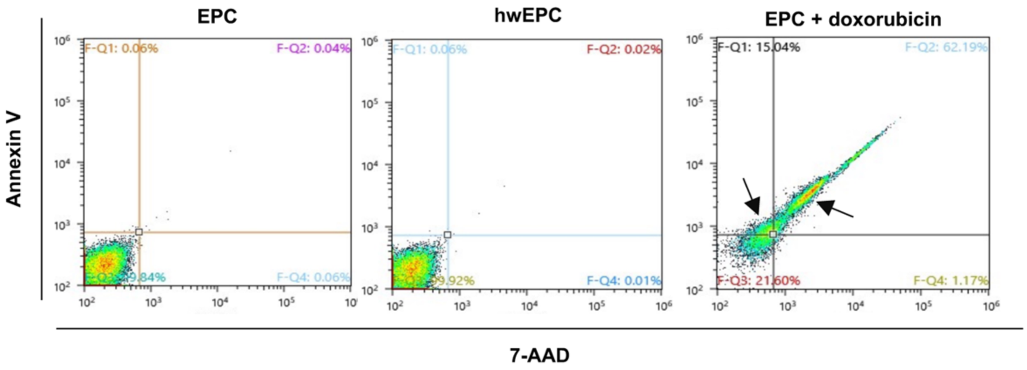

Supplementary Figure 3. The apoptosis of EPC and hwEPC was examined by staining a number of 10^6 cells with APC-Annexin V apoptosis detection kit with 7-AAD (640930, BioLegend). EPC treated with 0.25 µg/mL doxorubicin (Cayman Chemical) for 24 hours were used as the control groups with apoptosis. The experiments were performed in triplicate.

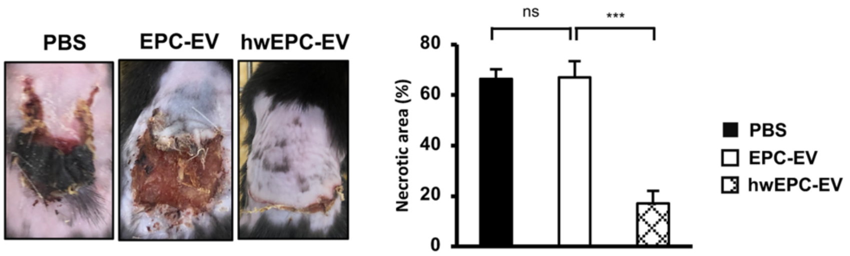

Supplementary Figure 4. The necrotic area of male ischemic flap mice with tail vein injection of EPC-EV or hwEPC-EV (n=3). ***p<0.001.

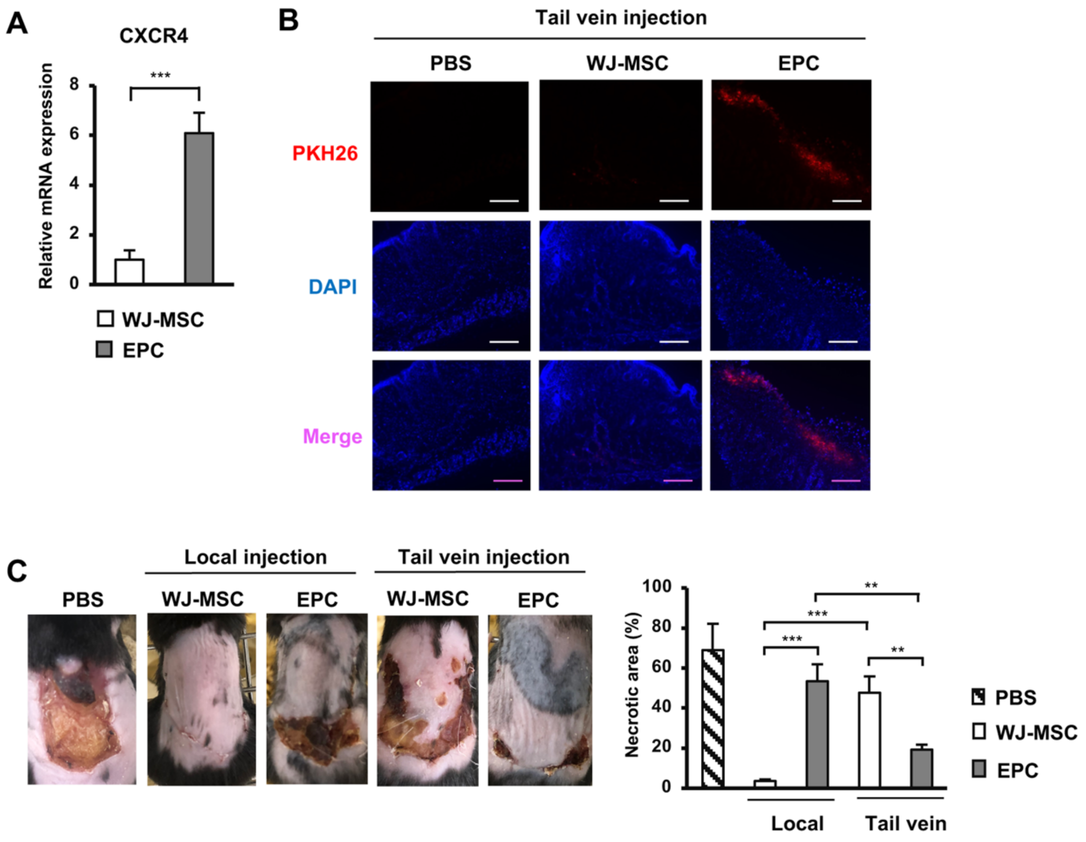

Supplementary Figure 5. A. CXCR4 expression in WJ-MSC and EPC (n=3). ***p<0.001. **B.** The fluorescence imaging analysis of PKH26-labeled EPC or WJ-MSC after 24 hours tail vein injection of a number of $5x10^5$ cells/mouse to the flap mice. Photos were captured under a fluorescence microscope at 20× magnification. Bars indicate 100 μm. (n=3). **C.** The necrotic area of ischemic flap mice locally injected or intravenously injected with WJ-MSC or EPC at a number of $5x10^5$ cells/mouse (n=3). All the above experiments were performed in triplicate. The data represent the mean ± SD. ***p<0.001, **p<0.01

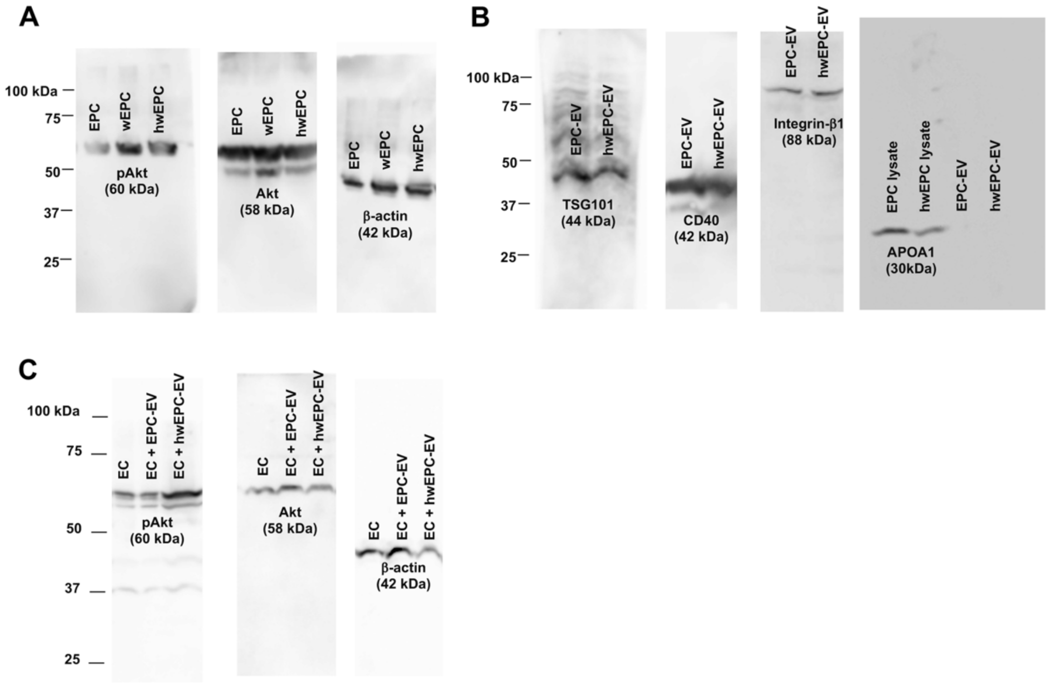

Supplementary Figure 6. A. Full length of Western blots shown in Figure 2G. **B.** Full length of Western blots shown in Figure 3B. **C.** Full length of Western blots shown in Figure 3J.
